# Cardiovascular disease essential medicines listing by countries: changes over time and association with health outcomes

**DOI:** 10.1186/s12872-024-04411-y

**Published:** 2025-01-27

**Authors:** Camila Heredia, Moizza Zia Ul Haq, Adelaide Buadu, Amal Rizvi, Aine Workentin, Navindra Persaud

**Affiliations:** 1 MAP Centre for Urban Health Solutions, Li Ka Shing Knowledge Institute, St. Michael’s Hospital, Unity Health Toronto, 80 Bond Street, Toronto, ON M5B 1X2 Canada; 2 Department of Family and Community Medicine, Faculty of Medicine, University of Toronto, Toronto, ON Canada

**Keywords:** Essential medicines, Cardiovascular disease, Ischemic heart disease, Cerebrovascular disease, Hypertensive heart disease, Amenable mortality

## Abstract

**Background:**

Since national essential medicine lists guide the procurement of medicines for populations in many countries, and cardiovascular diseases are the leading cause of death globally, including cardiovascular medicines on these lists can significantly impact healthcare outcomes.

**Methods:**

In this cross-sectional study, national essential medicines’ lists from 158 countries were analysed on whether or not they included medicines to treat ischemic heart disease, cerebrovascular disease, and hypertensive heart disease. A linear regression model was used to evaluate the association between countries’ coverage scores and amenable mortality.

**Results:**

Listing of cardiovascular disease treatment was associated with amenable mortality from hypertensive heart disease. Health expenditure per capita was also associated with amendable mortality due to ischemic heart disease, and hypertensive heart disease.

**Conclusions:**

Listing essential medicines for cardiovascular disease is an important aspect of healthcare quality that is associated with cardiovascular mortality.

**Supplementary Information:**

The online version contains supplementary material available at 10.1186/s12872-024-04411-y.

## Background

Cardiovascular disease remains the leading cause of death worldwide despite effective interventions [1]. For example, hypertension continues to be a significant yet preventable risk factor for cardiovascular disease events, contributing to 55% of deaths related to ischemic heart disease and 45% of deaths related to cerebrovascular disease [2]. Access to care and medicines varies significantly across different regions [1], and sometimes, limited accessibility and very high expenses pose significant obstacles to the utilization and compliance with essential treatments for cardiovascular diseases. For instance, a considerable portion of people in low-income and middle-income countries lack access to more than one blood pressure-lowering medicine [3]. Even when accessible, these medicines are frequently financially out of reach [3]. Such circumstances contribute to inadequate management of cardiovascular risk factors [3]. As the emergence of newer treatments for cardiovascular disease holds promise in substantially reducing cardiovascular morbidity and mortality rates, guaranteeing the affordability and accessibility of those medicines is also imperative.

Essential Medicine Lists (EMLs) represent a crucial component of national drug policies [4]. The World Health Organization (WHO), the specialized agency of the United Nations responsible for international public health, advocates for countries to maintain EMLs that prioritize treatments for prevalent health issues like cardiovascular disease. By serving as a guide, EMLs play a vital role in addressing the burden of non-communicable diseases [5]. 

There are usually opportunities for improvements in national EMLs as they may be outdated, either because they include obsolete medicines or because they exclude newer ones [6]. Moreover, the inclusion of a specific medication in EMLs is associated with its availability and affordability across both public and private health sectors [2]. 

The purpose of this study was to evaluate the relationship between the inclusion of essential medicines used to treat ischemic heart disease, cerebrovascular disease, and hypertensive heart disease, and the associated mortality rates measured by the HAQ (Healthcare Access and Quality) score. Additionally, we analyzed changes in the listing of these treatments over a six-year period.

## Methods

### Data sources

We employed the Global Essential Medicines (GEM) database of national EMLs that was updated in 2023. Briefly, the database was created by searching for national EMLs, having at least two researchers abstract data from each national EML and checking the data abstraction process. An algorithm was used to translate some medicine names and to assign ATC (Anatomical Therapeutic Chemical Classification) codes [7]. The database consists of a matrix listing each medicine and each country and indicates which countries list which medicines.

To identify medicines pertinent to the three specific conditions - ischemic heart disease, cerebrovascular disease, and hypertensive heart disease - we conducted a search for corresponding guidelines on the WHO website in November 2023. Three international guidelines distributed by the WHO were selected (1) Technical package for cardiovascular disease management in primary health care: Evidence-based treatment protocols 2018 [4], (2) Technical package for cardiovascular disease management in primary health care: Access to essential medicines and technology 2018 [8], and (3) Package of essential non-communicable disease interventions for primary health care 2020 [9]. 

We employed these guidelines, alongside reference to the WHO Model List 23rd edition [10], to identify medicines utilized in treating ischemic heart disease, cerebrovascular disease, and hypertensive heart disease. The guidelines were explored using the specific causes and their corresponding International Classification of Diseases 11th revision codes as provided by the HAQ score [11]. 

Data concerning population size was obtained from the United Nations [12], and data related to health expenditure was obtained from the Global Health Observatory [13], except for Somalia and Democratic People’s Republic of Korea [14, 15]. Most of the data pertained to the year 2023; if 2023 records were unavailable, information from the nearest available year to 2023 was accessed.

We employed the 2022 amenable mortality sub scores which were determined by analyzing age-standardized mortality rates related to ischemic heart disease, cerebrovascular disease, and hypertensive heart disease [11]. Amenable mortality has been defined as those premature deaths that should have not occurred in the presence of timely and effective health care [16]. 

### Data extraction

Employing the guidelines specified for ischemic heart disease, cerebrovascular disease, and hypertensive heart disease, medicines used to manage these conditions were extracted. Whenever a guideline specified a therapeutic category of medicines, that category was comprehensively expanded to include all medicines, as medicines falling within the same chemical subgroup might be regarded as therapeutically akin. The WHO Model List acknowledges the exchangeability of certain medicines within the same therapeutic class [10]. The ATC codes [7] were utilized to determine medicines belonging to the same therapeutic class. In instances where a therapeutic class was specified along with alternatives, solely those mentioned medicines were incorporated without expanding the therapeutic class. Medicines either listed directly on the WHO Model List or cited in guidelines referenced within the WHO Model List (in a practical form for the respective conditions or causes) and marked with a square box symbol were expanded. This expansion was based on the ATC code group, the chemical subgroup of the code, encompassing all medicines included in that therapeutic class. Medicines lacking the square box symbol were not expanded. In cases where specific equivalent medicines were specified, only those identified medicines were included. A medicine coverage score was formulated by summing the count of medicines included in a country’s national EML that also appeared on our list of medicines used to treat each of the three conditions.

### Data analysis

The analysis utilized Stata (16, StataCorp LLC, College Station, TX), with statistical significance set at a *p*-value ≤ 0.05. A linear regression model was fitted to assess the hypothesis regarding the relationship between the listing of medicines (measured as the medicine coverage score) and amenable mortality. In this analysis, the risk-standardized death rate from the HAQ dataset was the measure of amenable mortality, and the medicine coverage score served as the independent variable [11]. The regression results are presented for both unadjusted and adjusted models, with health expenditure and population size as pre-specified covariates that were included because they could be associated with both medicine coverage and amenable mortality.

## Results

We identified national EMLs and HAQ scores for 158 countries (Table 1). These countries were distributed across WHO regions as follows: Eastern Mediterranean (18 countries), Europe (32 countries), Africa (47 countries), the Americas (30 countries), South-East Asia (11 countries), and the Western Pacific (20 countries) [16]. According to the 2023 World Bank categorization, the countries included encompassed diverse income levels, comprising 26 low-income countries, 50 lower-middle-income countries, 51 upper-middle-income countries, and 31 high-income countries [18]. 

**Table 1:** Country characteristics. Note: income level was extracted from website in February 2024

Country	ISO code [23]	Geographic region [17]	Income group level [24]	Ischemic heart disease medicine coverage score	Cerebrovascular disease medicine coverage score	Hypertensive heart disease medicine coverage score	Health expenditure (2021) (US$ per capita) [13]	Population (2021) [12]	Year of NEML list
Afghanistan	AFG	Eastern Mediterranean	Low	29	20	18	81	40,099,462	2015
Albania	ALB	European	Upper middle	48	44	36	465	2,854,710	2022
Algeria	DZA	Africa	Lower middle	58	52	46	205	44,177,969	2023
Angola	AGO	Africa	Lower middle	31	23	21	64	34,503,774	2021
Antigua and Barbuda	ATG	The Americas	High	35	26	21	923	93,220	2022
Argentina	ARG	The Americas	Upper middle	14	12	11	1,045	45,276,780	2021
Armenia	ARM	European	Upper middle	39	28	24	613	2,790,974	2021
Australia	AUS	Western Pacific	High	59	54	44	7,055	25,921,089	2023
Bahrain	BHR	Eastern Mediterranean	High	46	36	32	1,146	1,463,266	2015
Bangladesh	BGD	South-East Asia	Lower middle	26	18	16	58	169,356,251	2019
Belarus	BLR	European	Upper middle	57	46	35	468	9,578,168	2021
Benin	BEN	Africa	Lower middle	38	28	21	35	12,996,895	2018
Bhutan	BTN	South-East Asia	Lower middle	37	28	22	120	777,487	2021
Bolivia (Plurinational State of)	BOL	The Americas	Lower middle	37	27	22	273	12,079,472	2022
Bosnia and Herzegovina	BIH	European	Upper middle	28	25	23	692	3,270,943	2019
Botswana	BWA	Africa	Upper middle	34	24	20	457	2,588,423	2012
Brazil	BRA	The Americas	Upper middle	34	27	20	761	214,326,223	2022
Bulgaria	BGR	European	Upper middle	63	61	53	1,040	6,885,868	2023
Burkina Faso	BFA	Africa	Low	39	31	23	57	22,100,684	2020
Burundi	BDI	Africa	Low	30	21	19	24	12,551,213	2022
Cabo Verde	CPV	Africa	Lower middle	32	23	18	248	587,925	2018
Cambodia	KHM	Western Pacific	Lower middle	25	16	15	122	16,589,024	2018
Cameroon	CMR	Africa	Lower middle	33	24	18	64	27,198,628	2022
Central African Republic	CAF	Africa	Low	29	22	18	43	5,457,155	2017
Chad	TCD	Africa	Low	40	30	24	36	17,179,740	2022
Chile	CHL	The Americas	High	34	24	22	1,518	19,493,185	2006
China	CHN	Western Pacific	Upper middle	45	37	30	671	1,425,893,465	2018
Colombia	COL	The Americas	Upper middle	75	68	58	558	51,516,562	2019
Comoros	COM	Africa	Lower middle	25	16	13	99	821,626	2014
Congo	COG	Africa	Lower middle	30	22	18	81	5,835,806	2019
Cooks Islands	COK	Western Pacific	Upper middle	20	22	18	737	17,003	2017
Costa Rica	CRI	The Americas	Upper middle	23	22	17	949	5,153,957	2019
Cote D’Ivoire	CIV	Africa	Lower middle	26	40	35	82	27,478,249	2020
Croatia	HRV	European	High	46	42	29	1,384	4,060,136	2022
Cuba	CUB	The Americas	Upper middle	35	25	23	1,186	11,256,373	2018
Czechia	CZE	European	High	73	64	56	2,499	10,510,751	2012
Democratic People’s Republic of Korea	PRK	South-East Asia	Low	21	14	13	0.5	25,971,909	2012
Democratic Republic of Congo	COD	Africa	Low	33	25	18	22	95,894,119	2020
Djibouti	DJI	Eastern Mediterranean	Lower middle	18	11	11	88	1,105,558	2007
Dominica	DMA	The Americas	Upper middle	35	26	21	482	72,413	2022
Dominican Republic	DOM	The Americas	Upper middle	39	31	26	417	11,117,874	2018
Ecuador	ECU	The Americas	Upper middle	35	27	19	494	17,797,737	2019
Egypt	EGY	Eastern Mediterranean	Lower middle	36	25	22	180	109,262,178	2018
El Salvador	SLV	The Americas	Upper middle	29	20	16	442	6,314,168	2020
Equatorial Guinea	GNQ	Africa	Upper middle	19	13	11	256	1,634,466	2012
Eritrea	ERI	Africa	Low	27	18	18	25	3,620,312	2010
Estonia	EST	European	High	53	50	42	2,095	1,328,701	2012
Eswatini	SWZ	Africa	Lower middle	27	20	16	280	1,192,271	2012
Ethiopia	ETH	Africa	Low	32	25	19	26	120,283,026	2020
Fiji	FJI	Western Pacific	Upper middle	26	17	15	250	924,610	2015
Gabon	GAB	Africa	Upper middle	16	10	8	234	2,341,179	2019
Gambia	GMB	Africa	Low	24	17	15	25	2,639,916	2019
Georgia	GEO	European	Upper middle	23	15	12	417	3,757,980	2007
Ghana	GHA	Africa	Lower middle	40	32	28	100	32,833,031	2017
Greece	GRC	European	High	83	70	61	1,846	10,445,365	2007
Grenada	GRD	The Americas	Upper middle	35	26	21	505	124,610	2022
Guatemala	GTM	The Americas	Upper middle	41	31	25	341	17,608,484	2021
Guinea	GIN	Africa	Lower middle	42	33	30	45	13,531,906	2021
Guinea-Bissau	GNB	Africa	Low	39	30	26	69	2,060,721	2020
Guyana	GUY	The Americas	High	37	27	21	471	804,567	2021
Haiti	HTI	The Americas	Lower middle	31	23	19	58	11,447,569	2020
Honduras	HND	The Americas	Lower middle	30	20	16	254	10,278,346	2018
Iceland	ISL	European	High	19	21	7	6,716	370,335	2022
India	IND	South-East Asia	Lower middle	34	26	18	74	1,407,563,842	2022
Indonesia	IDN	South-East Asia	Upper middle	34	25	21	161	273,753,191	2021
Iran (Islamic Republic of)	IRN	Eastern Mediterranean	Lower middle	60	53	37	393	87,923,433	2017
Iraq	IRQ	Eastern Mediterranean	Upper middle	46	39	29	249	43,533,593	2014
Ireland	IRL	European	High	56	54	45	6,764	4,986,526	2023
Jamaica	JAM	The Americas	Upper middle	45	36	29	372	2,827,695	2015
Japan	JPN	Western Pacific	High	4	1	0	4,347	124,612,531	2018
Jordan	JOR	Eastern Mediterranean	Lower middle	39	32	25	299	11,148,278	2021
Kazakhstan	KAZ	European	Upper middle	5	2	1	403	19,196,466	2020
Kenya	KEN	Africa	Lower middle	38	28	23	95	53,005,614	2019
Kiribati	KIR	Western Pacific	Lower middle	24	16	15	262	128,874	2009
Kyrgyzstan	KGZ	European	Lower middle	40	31	24	73	6,527,744	2009
Latvia	LVA	European	High	47	46	37	1,898	1,873,919	2023
Lebanon	LBN	Eastern Mediterranean	Lower middle	35	27	22	307	5,592,631	2018
Lesotho	LSO	Africa	Lower middle	24	15	14	115	2,281,455	2005
Liberia	LBR	Africa	Low	22	15	16	112	5,193,416	2022
Libya	LBY	Eastern Mediterranean	Upper middle	42	30	26	381	6,735,277	2019
Lithuania	LTU	European	High	52	45	43	1,859	2,786,651	2012
Madagascar	MDG	Africa	Low	32	20	16	18	28,915,653	2019
Malawi	MWI	Africa	Low	30	21	20	47	19,889,742	2015
Malaysia	MYS	Western Pacific	Upper middle	34	29	21	487	33,573,874	2023
Maldives	MDV	South-East Asia	Upper middle	62	55	40	1,039	521,458	2021
Mali	MLI	Africa	Low	34	24	20	40	21,904,983	2019
Malta	MLT	European	High	50	41	34	3,642	526,748	2022
Marshall Islands	MHL	Western Pacific	Upper middle	27	19	17	767	42,050	2007
Mauritania	MRT	Africa	Lower middle	37	27	23	89	4,614,974	2021
Mauritius	MUS	Africa	Upper middle	36	26	22	565	1,298,915	2022
Mexico	MEX	The Americas	Upper middle	55	50	36	611	126,705,138	2017
Mongolia	MNG	Western Pacific	Lower middle	45	35	29	316	3,347,783	2020
Montenegro	MNE	European	Upper middle	49	43	31	985	627,859	2020
Morocco	MAR	Eastern Mediterranean	Lower middle	36	26	20	221	37,076,585	2017
Mozambique	MOZ	Africa	Low	29	20	18	45	32,077,072	2017
Myanmar	MMR	South-East Asia	Lower middle	42	31	27	65	53,798,085	2016
Namibia	NAM	Africa	Upper middle	33	23	20	456	2,530,151	2016
Nauru	NRU	Western Pacific	High	27	17	15	1,530	12,512	2010
Nepal	NPL	South-East Asia	Lower middle	33	24	20	65	30,034,990	2021
Nicaragua	NIC	The Americas	Lower middle	30	21	19	198	6,850,540	2011
Niger	NER	Africa	Low	26	16	15	34	25,252,722	2018
Nigeria	NGA	Africa	Lower middle	42	34	29	84	213,401,323	2020
Niue	NIU	Western Pacific	Upper middle income	26	19	19	1,912	1,937	2006
North Macedonia	MKD	European	Upper middle income	26	17	15	560	2,103,330	2015
Oman	OMN	Eastern Mediterranean	High	62	56	45	853	4,520,471	2020
Pakistan	PAK	Eastern Mediterranean	Lower middle	47	42	30	43	231,402,117	2021
Palau	PLW	Western Pacific	Upper middle	34	25	20	2,045	18,024	2017
Panama	PAN	The Americas	High	4	1	0	1,415	4,351,267	2019
Paraguay	PRY	The Americas	Upper middle	33	23	18	479	6,703,799	2009
Peru	PER	The Americas	Upper middle	40	29	22	412	33,715,472	2018
Philippines	PHL	Western Pacific	Lower middle	49	39	33	203	113,880,328	2022
Poland	POL	European	High	52	46	39	1,159	38,307,726	2017
Portugal	PRT	European	High	10	8	7	2,747	10,290,103	2020
Republic of Korea	KOR	Western Pacific	High	11	9	7	3,260	51,830,139	2019
Republic of Moldova	MDA	European	Upper middle	36	26	20	410	3,061,507	2021
Romania	ROU	European	High	3	1	0	963	19,328,560	2021
Russian Federation	RUS	European	Upper middle	40	33	24	936	145,102,755	2019
Rwanda	RWA	Africa	Low	32	24	19	60	13,461,888	2022
Saint Kitts and Nevis	KNA	The Americas	High	35	26	21	1,114	47,607	2022
Saint Lucia	LCA	The Americas	Upper middle	35	26	21	585	179,652	2022
Saint Vincent and Grenadines	VCT	The Americas	Upper middle	35	26	21	448	104,332	2022
Sao Tome and Principe	STP	Africa	Lower middle	31	22	18	186	223,108	2022
Saudi Arabia	SAU	Eastern Mediterranean	High	56	50	42	1,442	35,950,396	2020
Senegal	SEN	Africa	Lower middle	30	19	16	71	16,876,720	2018
Serbia	SRB	European	Upper middle	66	55	45	919	7,296,769	2022
Seychelles	SYC	Africa	High	35	27	22	718	106,471	2022
Sierra Leone	SLE	Africa	Low	25	17	15	43	8,420,641	2021
Slovakia	SVK	European	High	58	53	42	1,685	5,447,622	2023
Slovenia	SVN	European	High	68	59	49	2,775	2,119,410	2017–2023
Solomon Islands	SLB	Western pacific	Lower middle	28	19	16	106	707,851	2017
Somalia	SOM	Eastern Mediterranean	Low	27	19	16	33	17,065,581	2019
South Africa	ZAF	Africa	Upper middle	20	13	11	584	59,392,255	2020–2021
South Sudan	SSD	Africa	Low	27	18	16	33	10,748,273	2018
Spain	ESP	European	High	5	3	3	3,234	47,486,935	2019
Sri Lanka	LKA	South-East Asia	Lower middle	26	19	17	166	21,773,441	2019
Sudan	SDN	Eastern Mediterranean	Low	52	41	36	22	45,657,202	2014
Suriname	SUR	The Americas	Upper middle	28	19	17	299	612,985	2022
Sweden	SWE	European	High	34	33	21	6,901	10,467,097	2023
Syrian Arab Republic	SYR	Eastern Mediterranean	Low	29	21	17	89	21,324,367	2019
Tajikistan	TJK	European	Lower middle	28	20	17	73	9,750,064	2009
Thailand	THA	South-East Asia	Upper middle	46	38	30	364	71,601,103	2021
Timor-Leste	TLS	South-East Asia	Lower middle	28	18	15	135	1,320,942	2015
Togo	TGO	Africa	Low	32	22	18	54	8,644,829	2012
Tonga	TON	Western Pacific	Upper middle	29	19	16	279	106,017	2007
Trinidad & Tobago	TTO	The Americas	High	42	34	28	1,125	1,525,663	2019
Tunisia	TUN	Eastern Mediterranean	Lower middle	68	55	50	265	12,262,946	2012
Tuvalu	TUV	Western Pacific	Upper middle	23	16	13	1,071	11,204	2010
Uganda	UGA	Africa	Low	34	24	20	43	45,853,778	2016
Ukraine	UKR	European	Lower middle	15	13	7	368	43,531,422	2017
United Republic of Tanzania	TZA	Africa	Lower middle	49	40	34	37	63,588,334	2021
Uruguay	URY	The Americas	High	47	37	31	1,620	3,426,260	2020
Uzbekistan	UZB	European	Lower middle	38	31	22	157	34,081,449	2021
Vanuatu	VUT	Western Pacific	Lower middle	19	13	11	133	319,137	2014
Venezuela (Bolivarian Republic of)	VEN	The Americas	Upper middle	30	21	17	160	28,199,867	2015
Viet Nam	VNM	Western Pacific	Lower middle	30	19	17	173	97,468,029	2018
Yemen	YEM	Eastern Mediterranean	Low	33	24	18	63	32,981,641	2019
Zambia	ZMB	Africa	Lower middle	36	28	26	75	19,473,125	2020
Zimbabwe	ZWE	Africa	Lower middle	35	28	23	63	15,993,524	2020

Overall, the most cited overall cardiovascular medicines included: morphine (listed by 153 countries), acetylsalicylic acid (listed by 151 countries), furosemide (listed by 148 countries), and spironolactone (listed by 147 countries).

### Ischemic heart disease

**Fig. 1:**
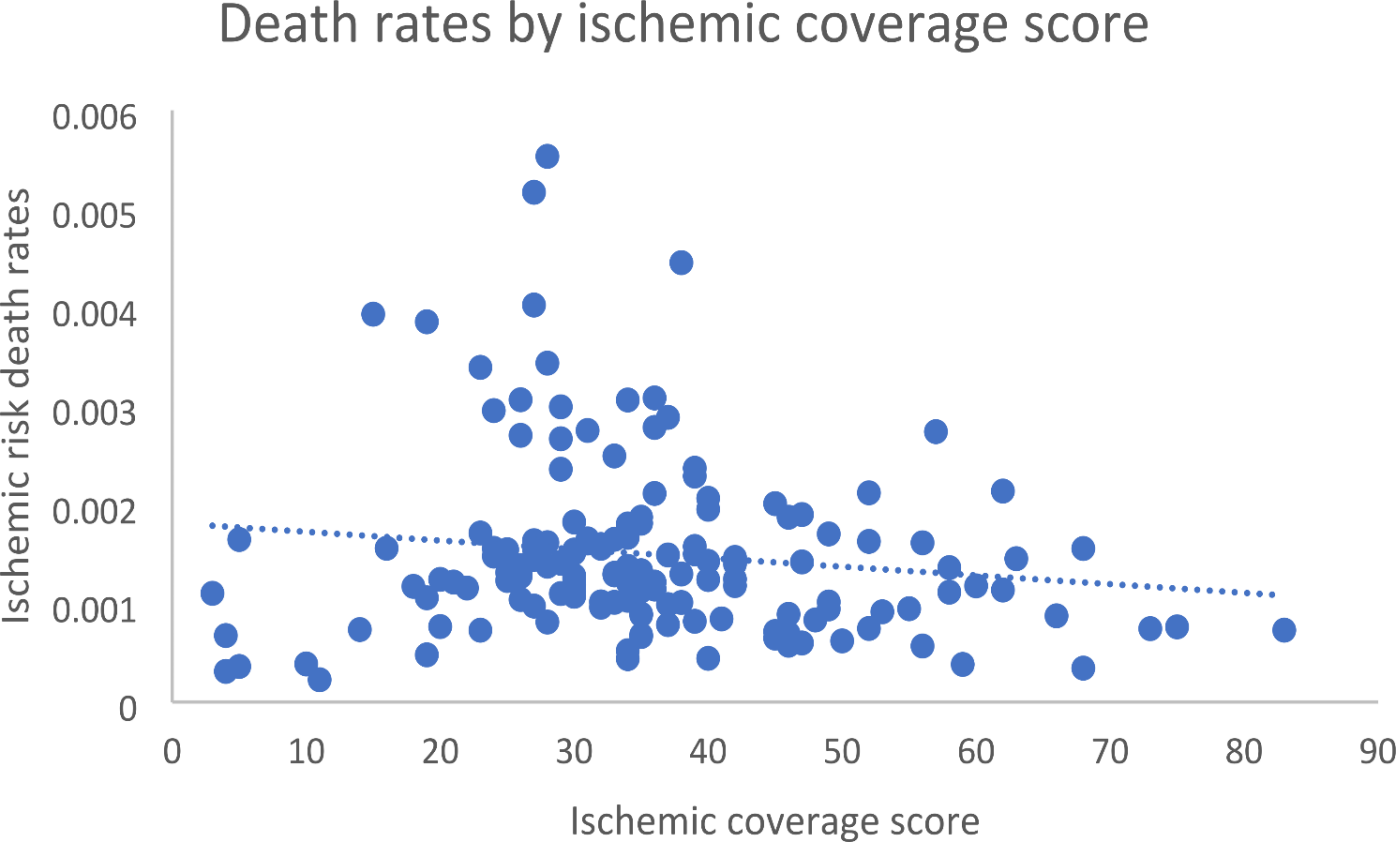
Ischemic death rates by coverage score

For ischemic heart disease, the range of medicine coverage scores spanned from 3 (Romania) to 83 (Greece), with a median of 34 (IQR: 27.25–42). Variables included the ischemic coverage score, health expenditure per capita (U$S), and population size. The unadjusted regression model showed listing ischemic heart disease medicines accounted for roughly 1.90% of the variation in amenable mortality and that the association between coverage and amenable mortality did not reach statistical significance (*p* = 0.084). The model including adjustments for population size and health expenditure explained 10.51% of the variation in amendable mortality. In this adjusted model, medicine coverage was not associated with amenable mortality (*p* = 0.182) compared to health expenditure, which was associated with the amenable mortality (*p* < 0.0001) (Table 2; Fig. 1).

**Table 2:** ^1^Ischemic heart disease: regression results

	Variable	Beta	95% CI lower bound	95% CI upper bound	*P* value
**Unadjusted**	Medicine coverage score	−0.00000884	-0.0000189	0.00000120	0.084
**Adjusted**	Medicine coverage score	-0.00000660	-0.0000163	0.00000312	0.182
	Health expenditure	-0.000000209	-0.000000318	-0.000000100	0.000
	Population	-0.000000000000350	-0.000000000000481	-0.00000000000118	0.407

R^2^ unadjusted = 0.0190 (F = 3.03, (df = 1), *p* = 0.0839). R^2^ adjusted = 0.1051 (F = 6.03, (df = 3), *p* = 0.0007)

B: unstandardized coefficient, CI: confidence interval

^1^ Results are extracted from linear regression models

### Cerebrovascular disease

**Fig. 2:**
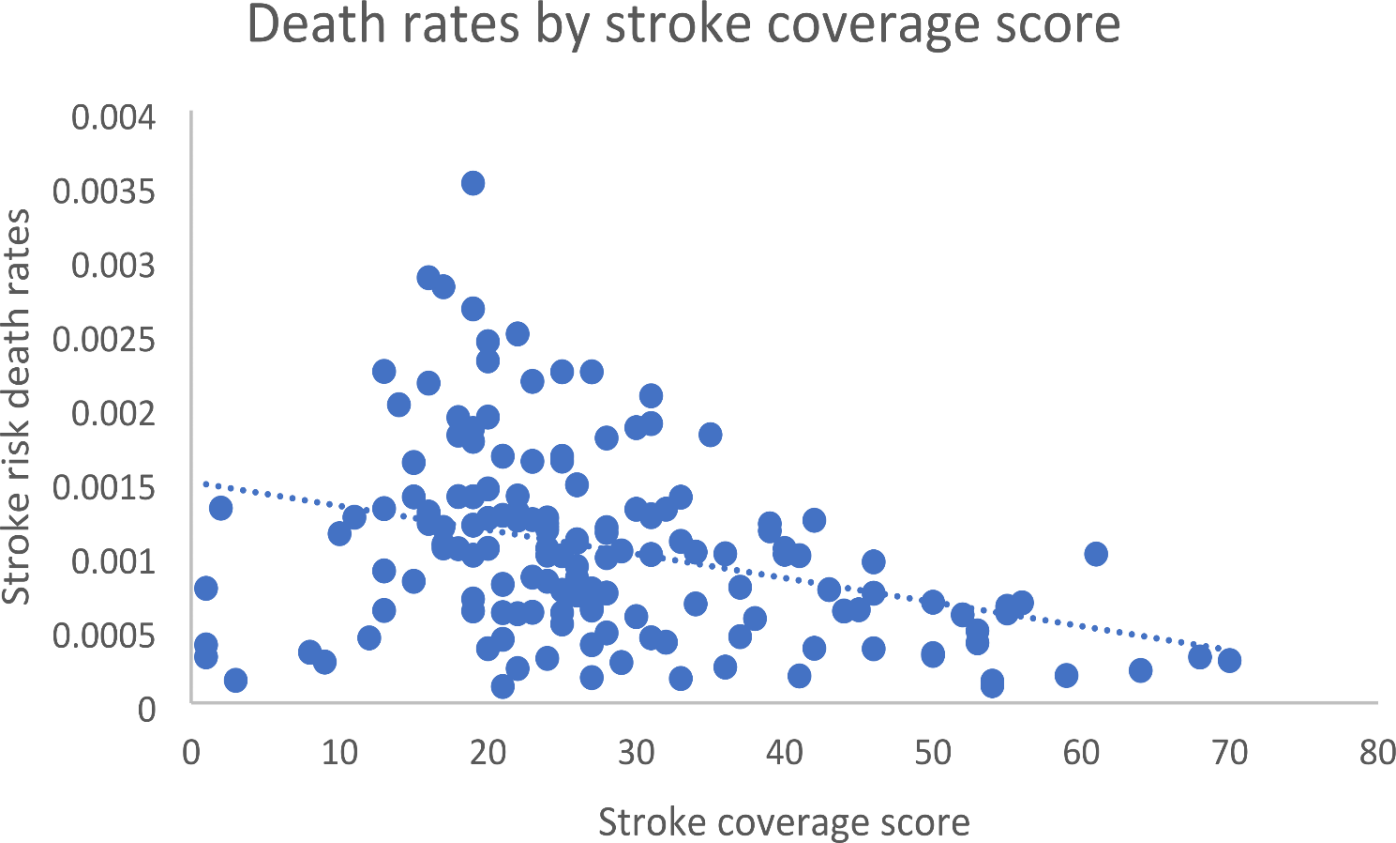
Stroke death rates by coverage score

For cerebrovascular disease, the medicine coverage scores ranged from 1 (Japan, Panama, Romania) to 70 (Greece), with a median of 25 (IQR: 19–33). Variables included the stroke coverage score, health expenditure per capita (U$S), and population size. The unadjusted regression model explained only 2.29% of the variation in amenable mortality and adjusting for population and health expenditure increased the amount of variation explained to just 3.55%. In the initial unadjusted regression, there was no association between medicine coverage score and HAQ score for cerebrovascular disease (*p* = 0.058). This was also the case in the adjusted model (*p* = 0.111). Population (*p* = 0.305) and health expenditure (*p* = 0.298) were also not associated with amenable mortality (Table 3; Fig. 2).

**Table 3:** ^1^Cerebrovascular disease: regression results

	Variable	Beta	95% CI lower bound	95% CI upper bound	*P* value
**Unadjusted**	Medicine coverage score	-0.00000710	-0.0000144	0.000000236	0.058
**Adjusted**	Medicine coverage score	-0.00000609	-0.0000136	0.00000142	0.111
	Health expenditure	-0.0000000429	-0.000000124	0.0000000382	0.298
	Population	-0.000000000000318	-0.000000000000928	0.000000000000293	0.305

R^2^ unadjusted = 0.0229 (F = 3.65, (df = 1), *p* = 0.0577). R^2^ adjusted = 0.0355 (F = 1.89, (df = 3), *p* = 0.1333)

B: unstandardized coefficient, CI: confidence interval

^1^ Results are extracted from linear regression models

### Hypertensive heart disease

**Fig. 3:**
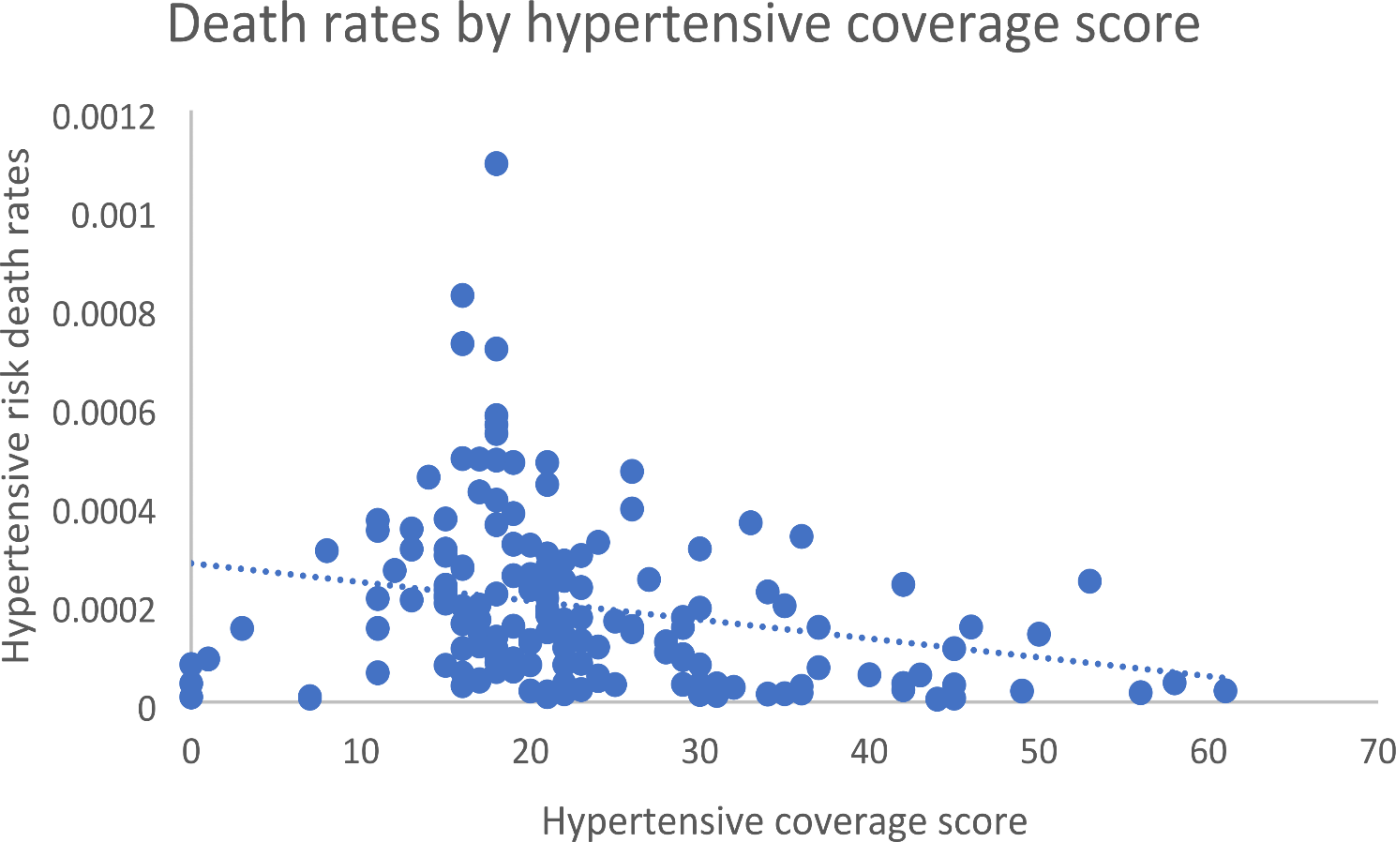
Hypertensive death rates by coverage score

For hypertensive heart disease, the medicine coverage scores range from 0 (Japan, Panama, Romania) to 61 (Greece), with a median of 20.5 (IQR: 16.25–28). Variables included the hypertensive coverage score, health expenditure per capita (U$S), and population size. The unadjusted model accounted for little variation in the amenable mortality (5.89%), compared to the adjusted model (18.87%). There was an association between medicine coverage score and amenable mortality in the unadjusted (*p* = 0.002) and adjusted (*p* = 0.015) models. Health expenditure (*p* = 0.000) was also associated with amenable mortality, compared to population (*p* = 0.268), which was not (Table 4; Fig. 3). Examples of countries with lower HAQ scores include Central African Republic, Somalia, and Chad, and have hypertensive medicine coverage scores of 18, 16, and 24 respectively. These three countries did not list 55 hypertensive medicines such as carvedilol, diltiazem, lercanidipine, nitroprusside, prazosin, and rosuvastatin that are all medicines commonly listed by other countries.

**Table 4:** ^1^Hypertensive heart disease: regression results

	Variable	Beta	95% CI lower bound	95% CI upper bound	*P* value
**Unadjusted** **Adjusted**	Medicine coverage score	-0.00000381	-0.00000623	-0.00000140	0.002
	Medicine coverage score	-0.00000286	-0.000000571	-0.00000514	0.015
	Health expenditure	-0.0000000507	-0.0000000722	-0.0000000302	0.000
	Population	-0.0000000000000872	-0.0000000000000678	-0.000000000000242	0.268

R^2^ unadjusted = 0.0589 (F = 9.76 (df = 1), *p* = 0.0021). R^2^ adjusted = 0.1887 (F = 11.94, (df = 3), *p* = 0.000)

B: unstandardized coefficient, CI: confidence interval

^1^ Results are extracted from linear regression models

## Discussion

Listing of cardiovascular disease treatment is associated with amenable mortality from hypertensive heart disease, but not for ischemic or cerebrovascular disease. Health expenditure per capita was associated with amendable ischemic and hypertensive mortality.

Given that expenses for cardiovascular disease treatment surpass the per capita health expenditure in many low to middle-income countries [19], prioritized access to cardiovascular disease treatments alongside improvements in care may be beneficial in many countries [20]. The benefits of prioritized access to cardiovascular disease treatments may be greatest in low- and middle-income countries [21]. 

Of course, medicine access is only one important aspect of the care pathways; a cross-sectional study in 44 low and middle-income countries highlighted the need for designing and implementing health policies for hypertension in health systems where their performance tend to be poor, with less than 50% of people with hypertension being diagnosed and less than a third receiving pharmacological treatment [22]. 

### Strengths and limitations

This is the largest study of cardiovascular disease essential medicines as far as we know. Causation should not be inferred from a cross-sectional study. Mortality-related data is estimate. The scoring system does not consider therapeutically interchangeable medicines within a class; theoretically, the presence of just one medicine in a class might suffice, rendering others redundant. There might also exist other potentially relevant covariates, such as population insurance coverage, lifestyle factors, or healthcare infrastructure. The presence of a medicine in the EML does not mean its accessibility and affordability, as some countries have public and private health sectors coexisting. Despite the limitations associated with developing a medicine coverage score, our methodology enabled the derivation of an overarching score for cross-country comparisons. Our study employed data used to estimate the HAQ score for countries and that dataset has limitation [11]. 

## Conclusions

In conclusion, these findings suggest that the listing of cardiovascular disease treatments appears to specifically impact mortality rates amenable to hypertensive heart disease treatment, while not significantly influencing outcomes related to ischemic or cerebrovascular disease. Additionally, higher health expenditure per capita is associated with reduced amenable mortality for both ischemic and hypertensive heart diseases. These results emphasize the importance of targeted health investments and the potential for improved outcomes in specific cardiovascular conditions, highlighting the nuanced role of essential medicines for cardiovascular disease and healthcare allocation in reducing disease-specific mortality rates.

Future work should delineate the contributions of types of medicines or specific medicines in addressing cardiovascular mortality.

## Electronic supplementary material

Below is the link to the electronic supplementary material.

Supplementary Material 1

## Data Availability

The datasets used and/or analysed during the current study are available from the corresponding author on reasonable request.

## Abbreviations

ATC: Anatomical Therapeutic Chemical Classification
GEM: Global Essential Medicines
HAQ: Healthcare Access and Quality
EML: Essential Medicine List
WHO: World Health Organization
