# Supplementary material for: Cardiovascular disease essential medicines listing by countries: changes over time and association with health outcomes
Source: BMC Cardiovasc Disord. 2025 Jan 27;25:50. doi: 10.1186/s12872-024-04411-y (PMC11771043; doi:10.1186/s12872-024-04411-y)
Supplement: Supplementary file 1 — Supplementary Material 1 [file 12872_2024_4411_MOESM1_ESM.docx]

**Additional file 1.** List of cardiovascular medicines

*Medicines for Ischemic heart disease*

| **Medicine Name** | **Primary ATC Code** |
| --- | --- |
| Acebutolol | C07AB04 |
| Acenocoumarol | B01AA07 |
| Acetylsalicylic acid | B01AC06 |
| Adenosine  Alteplase | C01EB10  B01AD02 |
| Amiloride  Amiodarone | C03DB01  C01BD01 |
| Amlodipine | C08CA01 |
| Atenolol | C07AB03 |
| Atorvastatin | C10AA05 |
| Benazepril | C09AA07 |
| Bendrofluazide (Bendroflumethiazide) | C03AA01 |
| Bepridil  Betaxolol | C08EA02  C07AB05 |
| Bisoprolol | C07AB07 |
| Bumetanide | C03CA02 |
| Candesartan | C09CA06 |
| Captopril | C09AA01 |
| Carteolol  Carvedilol | C07AA15  C07AG02 |
| Celiprolol | C07AB08 |
| Chlorothiazide | C03AA04 |
| Chlortalidone (Chlorthalidone) | C03BA04 |
| Cilazapril | C09AA08 |
| Cilnidipine | C08CA14 |
| Clopidogrel | B01AC04 |
| Cyclopenthiazide | C03AA07 |
| Dalteparin | B01AB04 |
| Delapril | C09AA12 |
| Digoxin | C01AA05 |
| Diltiazem  Dobutamine  Dopamine | C05AE03  C01CA07  C01CA04 |
| Drotrecogin alfa | B01AD10 |
| Enalapril | C09AA02 |
| Enoxaparin | B01AB05 |
| Epinephrine (Adrenaline) | C01CA24 |
| Eplerenone | C03DA04 |
| Eprosartan | C09CA02 |
| Esmolol | C07AB09 |
| Ethyl biscoumacetate | B01AA08 |
| Felodipine | C08CA02 |
| Fibrinolysin | B01AD05 |
| Fluindione | B01AA12 |
| Fluvastatin | C10AA04 |
| Fosinopril | C09AA09 |
| Furosemide | C03CA01 |
| Heparin | B01AB01 |
| Hydrochlorothiazide | C03AA03 |
| Hydromorphone | N02AA03 |
| Imidapril | C09AA16 |
| Indapamide | C03BA11 |
| Irbesartan | C09CA04 |
| Isosorbide dinitrate | C01DA08 |
| Isosorbide mononitrate | C01DA14 |
| Isradipine | C08CA03 |
| Ivabradine  Labetalol  Lacidipine | C01EB17  C07AG01  C08CA09 |
| Lercanidipine | C08CA13 |
| Levamlodipine  Lidocaine (Lignocaine, Xylocaine) | C08CA17  C05AD01 |
| Lisinopril | C09AA03 |
| Losartan | C09CA01 |
| Lovastatin | C10AA02 |
| Manidipine | C08CA11 |
| Methylchlothiazide  Methyldopa | C03AA08  C02AB01 |
| Metolazone  Metoprolol | C03BA08  C07AB02 |
| Moexipril | C09AA13 |
| Morphine | N02AA01 |
| Nadolol  Nadroparin | C07AA12  B01AB06 |
| Nebivolol | C07AB12 |
| Nicardipine | C08CA04 |
| Nifedipine | C08CA05 |
| Nilvadipine | C08CA10 |
| Nimodipine | C08CA06 |
| Nisoldipine | C08CA07 |
| Nitrendipine | C08CA08 |
| Nitroglycerin (Glyceryl trinitrate) | C01DA02 |
| Nitroprusside | C02DD01 |
| Olmesartan | C09CA08 |
| Oxygen | V03AN01 |
| Perindopril | C09AA04 |
| Phenprocoumon | B01AA04 |
| Pindolol  Pitavastatin | C07AA03  C10AA08 |
| Pravastatin | C10AA03 |
| Prazosin  Propranolol | C02CA01  C07AA05 |
| Quinapril | C09AA06 |
| Ramipril | C09AA05 |
| Ranolazine  Reteplase | C01EB18  B01AD07 |
| Rosuvastatin | C10AA07 |
| Simvastatin | C10AA01 |
| Sotalol  Spirapril | C07AA07  C09AA11 |
| Spironolactone | C03DA01 |
| Streptokinase* | B01AD01 |
| Telmisartan | C09CA07 |
| Tenecteplase | B01AD11 |
| Torasemide | C03CA04 |
| Trandolapril | C09AA10 |
| Urokinase | B01AD04 |
| Valsartan | C09CA03 |
| Verapamil | C08DA01 |
| Warfarin | B01AA03 |
| Zofenopril | C09AA15 |

*An exception to the rules stated in the methods was made for streptokinase, it is not denoted with a square box on the WHO Model List however, it was expanded. Two clinicians (NP, DM) agreed that it was worth expanding because all medicines in the therapeutic class were felt to be equivalent and widely used.

*Medicines for Cerebrovascular disease*

| **Medicine Name** | **Primary ATC Code** |
| --- | --- |
| Acebutolol | C07AB04 |
| Acenocoumarol | B01AA07 |
| Acetylsalicylic acid | B01AC06 |
| Alteplase | B01AD02 |
| Amiloride  Amiodarone | C03DB01  C01BD01 |
| Amlodipine | C08CA01 |
| Apixaban | B01AF02 |
| Atenolol | C07AB03 |
| Atorvastatin | C10AA05 |
| Azilsartan  Benazepril | C09CA09  C09AA07 |
| Bendrofluazide (Bendroflumethiazide) | C03AA01 |
| Betaxolol | C07AB05 |
| Bisoprolol | C07AB07 |
| Bumetanide | C03CA02 |
| Candesartan | C09CA06 |
| Captopril | C09AA01 |
| Carvedilol | C07AG02 |
| Celiprolol | C07AB08 |
| Chlorothiazide | C03AA04 |
| Chlortalidone (Chlorthalidone) | C03BA04 |
| Cilazapril | C09AA08 |
| Cilnidipine | C08CA14 |
| Clopidogrel | B01AC04 |
| Cyclopenthiazide | C03AA07 |
| Dabigatran | B01AE07 |
| Dalteparin | B01AB04 |
| Delapril | C09AA12 |
| Digoxin | C01AA05 |
| Diltiazem  Dipyridamole | C05AE03  B01AC07 |
| Drotrecogin alfa | B01AD10 |
| Edoxaban  Enalapril | B01AF03  C09AA02 |
| Enoxaparin | B01AB05 |
| Eplerenone | C03DA04 |
| Eprosartan | C09CA02 |
| Esmolol | C07AB09 |
| Ethyl biscoumacetate | B01AA08 |
| Felodipine | C08CA02 |
| Fibrinolysin | B01AD05 |
| Fluindione | B01AA12 |
| Fluvastatin | C10AA04 |
| Fosinopril | C09AA09 |
| Furosemide | C03CA01 |
| Heparin | B01AB01 |
| Hydrochlorothiazide | C03AA03 |
| Imidapril | C09AA16 |
| Indapamide | C03BA11 |
| Irbesartan | C09CA04 |
| Isradipine | C08CA03 |
| Labetalol  Lacidipine | C07AG01  C08CA09 |
| Landiolol | C07AB14 |
| Lercanidipine | C08CA13 |
| Lisinopril | C09AA03 |
| Losartan | C09CA01 |
| Lovastatin | C10AA02 |
| Manidipine | C08CA11 |
| Methylchlothiazide | C03AA08 |
| Metolazone  Metoprolol | C03BA08  C07AB02 |
| Moexipril | C09AA13 |
| Nadolol  Nadroparin | C07AA12  B01AB06 |
| Nebivolol | C07AB12 |
| Nicardipine | C08CA04 |
| Nifedipine | C08CA05 |
| Nilvadipine | C08CA10 |
| Nimodipine | C08CA06 |
| Nisoldipine | C08CA07 |
| Nitrendipine | C08CA08 |
| Nitroprusside | C02DD01 |
| Olmesartan | C09CA08 |
| Perindopril | C09AA04 |
| Phenprocoumon | B01AA04 |
| Pindolol  Pitavastatin | C07AA03  C10AA08 |
| Prasugrel  Pravastatin | B01AC22  C10AA03 |
| Prazosin  Propranolol | C02CA01  C07AA05 |
| Quinapril | C09AA06 |
| Ramipril | C09AA05 |
| Reteplase | B01AD07 |
| Rivaroxaban | B01AF01 |
| Rosuvastatin | C10AA07 |
| Simvastatin | C10AA01 |
| Spirapril | C09AA11 |
| Spironolactone | C03DA01 |
| Streptokinase* | B01AD01 |
| Telmisartan | C09CA07 |
| Tenecteplase | B01AD11 |
| Ticagrelor  Tirofiban  Torasemide | B01AC24  B01AC17  C03CA04 |
| Trandolapril | C09AA10 |
| Urokinase | B01AD04 |
| Valsartan | C09CA03 |
| Verapamil | C08DA01 |
| Warfarin | B01AA03 |
| Zofenopril | C09AA15 |

*An exception to the rules stated in the methods was made for streptokinase, it is not denoted with a square box on the WHO Model List however, it was expanded. Two clinicians (NP, DM) agreed that it was worth expanding because all medicines in the therapeutic class were felt to be equivalent and widely used.

*Medicines for Hypertensive heart disease*

| **Medicine Name** | **Primary ATC Code** |
| --- | --- |
| Acebutolol | C07AB04 |
| Aliskiren  Amiloride | C09XA02  C03DB01 |
| Amlodipine  Atenolol  Atorvastatin | C08CA01  C07AB03  C10AA05 |
| Azilsartan | C09CA09 |
| Benazepril | C09AA07 |
| Bendrofluazide (Bendroflumethiazide) | C03AA01 |
| Bepridil  Betaxolol | C08EA02  C07AB05 |
| Bisoprolol | C07AB07 |
| Bumetanide | C03CA02 |
| Candesartan | C09CA06 |
| Captopril | C09AA01 |
| Carteolol  Carvedilol | C07AA15  C07AG02 |
| Celiprolol | C07AB08 |
| Chlorothiazide | C03AA04 |
| Chlortalidone (Chlorthalidone) | C03BA04 |
| Cilazapril | C09AA08 |
| Cilnidipine | C08CA14 |
| Cyclopenthiazide | C03AA07 |
| Delapril | C09AA12 |
| Digoxin | C01AA05 |
| Diltiazem | C05AE03 |
| Dopamine  Enalapril | C01CA04  C09AA02 |
| Eplerenone | C03DA04 |
| Eprosartan | C09CA02 |
| Esmolol | C07AB09 |
| Felodipine | C08CA02 |
| Fluvastatin | C10AA04 |
| Fosinopril | C09AA09 |
| Furosemide | C03CA01 |
| Hydralazine | C02DB02 |
| Hydrochlorothiazide | C03AA03 |
| Imidapril | C09AA16 |
| Indapamide | C03BA11 |
| Irbesartan | C09CA04 |
| Isradipine | C08CA03 |
| Labetalol  Lacidipine | C07AG01  C08CA09 |
| Lercanidipine | C08CA13 |
| Levamlodipine  Lisinopril | C08CA17  C09AA03 |
| Losartan | C09CA01 |
| Lovastatin | C10AA02 |
| Manidipine | C08CA11 |
| Methylchlothiazide | C03AA08 |
| Methyldopa | C02AB01 |
| Metolazone | C03BA08 |
| Metoprolol  Moexipril | C07AB02  C09AA13 |
| Nadolol  Nebivolol | C07AA12  C07AB12 |
| Nicardipine | C08CA04 |
| Nifedipine | C08CA05 |
| Nilvadipine | C08CA10 |
| Nimodipine | C08CA06 |
| Nisoldipine | C08CA07 |
| Nitrendipine | C08CA08 |
| Nitroprusside | C02DD01 |
| Olmesartan | C09CA08 |
| Perindopril | C09AA04 |
| Pindolol  Pitavastatin | C07AA03  C10AA08 |
| Pravastatin | C10AA03 |
| Prazosin  Propranolol  Quinapril | C02CA01  C07AA05  C09AA06 |
| Ramipril | C09AA05 |
| Rosuvastatin | C10AA07 |
| Simvastatin | C10AA01 |
| Sotalol  Spirapril | C07AA07  C09AA11 |
| Spironolactone | C03DA01 |
| Telmisartan | C09CA07 |
| Torasemide | C03CA04 |
| Trandolapril | C09AA10 |
| Valsartan | C09CA03 |
| Verapamil  Zofenopril | C08DA01  C09AA15 |
|  |  |
